# Supplementary material for: Mechanisms governing the pioneering and redistribution capabilities of the non-classical pioneer PU.1
Source: Nat Commun. 2020 Jan 21;11:402. doi: 10.1038/s41467-019-13960-2 (PMC6972792; doi:10.1038/s41467-019-13960-2)
Supplement: Supplementary file 7 — Source data [file 41467_2019_13960_MOESM7_ESM.zip › Source_Data/FigureS4/FigureS4G_motifAnnotation/bound.singleMotif.PU1notmasked/knownResults.html]

/misc/data/analysis/project\_PU1/CTV1/motifs/basic/PU1.ntag15.filtered.singleMotif.PU1notmasked - Homer Known Motif Enrichment Results


# Homer Known Motif Enrichment Results (/misc/data/analysis/project\_PU1/CTV1/motifs/basic/PU1.ntag15.filtered.singleMotif.PU1notmasked)

Homer *de novo* Motif Results  
Gene Ontology Enrichment Results  
Known Motif Enrichment Results (txt file)  
Total Target Sequences = 22478, Total Background Sequences = 26477

|  |  |  |  |  |  |  |  |  |  |  |  |
| --- | --- | --- | --- | --- | --- | --- | --- | --- | --- | --- | --- |
| Rank | Motif | Name | P-value | log P-pvalue | q-value (Benjamini) | # Target Sequences with Motif | % of Targets Sequences with Motif | # Background Sequences with Motif | % of Background Sequences with Motif | Motif File | SVG |
| 1 | C G T A T A C G T C G A A C T G A C T G C G T A C G T A T A C G A G C T T A C G | PU.1(ETS)/ThioMac-PU.1-ChIP-Seq(GSE21512)/Homer | 1e-4393 | -1.012e+04 | 0.0000 | 14188.0 | 63.12% | 1519.8 | 5.74% | motif file (matrix) | svg |
| 2 | C G T A C T G A C G T A C T A G T C G A C T A G A C T G C G T A C G T A T A C G A G C T A T C G | SpiB(ETS)/OCILY3-SPIB-ChIP-Seq(GSE56857)/Homer | 1e-3731 | -8.593e+03 | 0.0000 | 11601.0 | 51.61% | 786.6 | 2.97% | motif file (matrix) | svg |
| 3 | T C G A T A G C T G C A A C T G A C T G C G T A C G T A C T A G G A C T T A C G | ETS1(ETS)/Jurkat-ETS1-ChIP-Seq(GSE17954)/Homer | 1e-2561 | -5.898e+03 | 0.0000 | 12409.0 | 55.21% | 2838.1 | 10.71% | motif file (matrix) | svg |
| 4 | G C T A A G T C T A C G T G C A A T C G T C A G G C T A T C G A T C A G A G C T | ELF5(ETS)/T47D-ELF5-ChIP-Seq(GSE30407)/Homer | 1e-2313 | -5.328e+03 | 0.0000 | 10957.0 | 48.75% | 2215.2 | 8.36% | motif file (matrix) | svg |
| 5 | C G T A T A G C T A G C T G C A A C T G C T A G C G T A C G T A T C A G G A C T | EHF(ETS)/LoVo-EHF-ChIP-Seq(GSE49402)/Homer | 1e-2165 | -4.986e+03 | 0.0000 | 12915.0 | 57.46% | 4010.9 | 15.14% | motif file (matrix) | svg |
| 6 | A T G C A G T C C T G A A G T C C G A T A C G T A G T C A G T C A C G T A T C G G A C T A C G T | Etv2(ETS)/ES-ER71-ChIP-Seq(GSE59402)/Homer(0.967) | 1e-2078 | -4.785e+03 | 0.0000 | 10992.0 | 48.90% | 2652.6 | 10.01% | motif file (matrix) | svg |
| 7 | A G T C C T G A A G T C C G A T C A G T G A T C A T G C A C T G A T C G G A C T | Fli1(ETS)/CD8-FLI-ChIP-Seq(GSE20898)/Homer | 1e-2070 | -4.768e+03 | 0.0000 | 11199.0 | 49.82% | 2819.3 | 10.64% | motif file (matrix) | svg |
| 8 | T C G A C T G A T A G C T G A C T C A G T C A G C G T A C G T A T C A G A G C T | ETV1(ETS)/GIST48-ETV1-ChIP-Seq(GSE22441)/Homer | 1e-2061 | -4.747e+03 | 0.0000 | 12341.0 | 54.91% | 3741.5 | 14.12% | motif file (matrix) | svg |
| 9 | T C G A T A G C G T C A A C T G A C T G C G T A C G T A C T A G A G C T T C A G | ERG(ETS)/VCaP-ERG-ChIP-Seq(GSE14097)/Homer | 1e-1995 | -4.596e+03 | 0.0000 | 13282.0 | 59.09% | 4715.5 | 17.80% | motif file (matrix) | svg |
| 10 | T C G A T C G A T A G C G T A C T C A G T A C G C G T A C G T A T C A G A G C T | GABPA(ETS)/Jurkat-GABPa-ChIP-Seq(GSE17954)/Homer | 1e-1709 | -3.937e+03 | 0.0000 | 9610.0 | 42.75% | 2360.0 | 8.91% | motif file (matrix) | svg |
| 11 | C G T A T G A C T A G C T G C A A C T G A C T G C G T A C G T A T C A G G A C T | ELF3(ETS)/PDAC-ELF3-ChIP-Seq(GSE64557)/Homer | 1e-1661 | -3.826e+03 | 0.0000 | 9154.0 | 40.73% | 2138.3 | 8.07% | motif file (matrix) | svg |
| 12 | T G C A C T G A A T G C G T C A A C T G A C T G C G T A C G T A C T A G A G C T | Ets1-distal(ETS)/CD4+-PolII-ChIP-Seq(Barski\_et\_al.)/Homer | 1e-1113 | -2.564e+03 | 0.0000 | 5284.0 | 23.51% | 782.0 | 2.95% | motif file (matrix) | svg |
| 13 | T C G A A G C T A C G T A C G T A G T C A G T C A C G T A T C G G A C T A T C G | EWS:ERG-fusion(ETS)/CADO\_ES1-EWS:ERG-ChIP-Seq(SRA014231)/Homer | 1e-952 | -2.192e+03 | 0.0000 | 6974.0 | 31.03% | 2128.3 | 8.03% | motif file (matrix) | svg |
| 14 | C T G A T G C A T A G C T G A C T A C G T C A G C T G A G C T A T C A G G A C T | ELF1(ETS)/Jurkat-ELF1-ChIP-Seq(SRA014231)/Homer | 1e-880 | -2.027e+03 | 0.0000 | 5185.0 | 23.07% | 1123.7 | 4.24% | motif file (matrix) | svg |
| 15 | T G C A T C G A T A G C G T A C T C A G C T A G G T C A G C T A T C A G G A C T | ETS(ETS)/Promoter/Homer | 1e-765 | -1.764e+03 | 0.0000 | 4054.0 | 18.04% | 701.4 | 2.65% | motif file (matrix) | svg |
| 16 | T C G A T A G C G T C A A C T G C T A G C G T A C G A T A C T G A C G T A C T G A C T G A C G T | ETS:RUNX(ETS,Runt)/Jurkat-RUNX1-ChIP-Seq(GSE17954)/Homer | 1e-562 | -1.296e+03 | 0.0000 | 2445.0 | 10.88% | 248.6 | 0.94% | motif file (matrix) | svg |
| 17 | T G A C C T A G T C A G G T C A C G T A T C A G C G A T T C A G T C G A T G C A C T G A T A G C | PU.1-IRF(ETS:IRF)/Bcell-PU.1-ChIP-Seq(GSE21512)/Homer | 1e-552 | -1.273e+03 | 0.0000 | 8682.0 | 38.63% | 4837.9 | 18.26% | motif file (matrix) | svg |
| 18 | C T G A A T G C C G T A A C G T A G T C A G T C A C G T A C T G A T C G G C A T | SPDEF(ETS)/VCaP-SPDEF-ChIP-Seq(SRA014231)/Homer | 1e-358 | -8.261e+02 | 0.0000 | 5108.0 | 22.73% | 2504.3 | 9.45% | motif file (matrix) | svg |
| 19 | G A T C T C G A A G T C C G A T C G A T A G T C A T G C A C T G A T C G G A C T | Elk1(ETS)/Hela-Elk1-ChIP-Seq(GSE31477)/Homer | 1e-342 | -7.894e+02 | 0.0000 | 3142.0 | 13.98% | 1067.5 | 4.03% | motif file (matrix) | svg |
| 20 | T G C A C T G A A G T C G T C A A C T G A C T G C G T A C G T A C T G A A G C T | EWS:FLI1-fusion(ETS)/SK\_N\_MC-EWS:FLI1-ChIP-Seq(SRA014231)/Homer | 1e-291 | -6.709e+02 | 0.0000 | 3514.0 | 15.63% | 1500.0 | 5.66% | motif file (matrix) | svg |
| 21 | G A T C C T G A A G T C C G A T C G A T G A T C A G T C A C T G A T C G A G C T | Elk4(ETS)/Hela-Elk4-ChIP-Seq(GSE31477)/Homer | 1e-225 | -5.189e+02 | 0.0000 | 2578.0 | 11.47% | 1038.6 | 3.92% | motif file (matrix) | svg |
| 22 | T C A G T C A G G C T A C G T A T A C G G A C T T C A G T C G A C T G A C G T A T A C G G A C T | IRF8(IRF)/BMDM-IRF8-ChIP-Seq(GSE77884)/Homer | 1e-188 | -4.337e+02 | 0.0000 | 2430.0 | 10.81% | 1062.6 | 4.01% | motif file (matrix) | svg |
| 23 | C T A G C T A G C G T A C G T A T A C G C G A T C T A G C T G A C T G A C G T A T A C G G A C T | PU.1:IRF8(ETS:IRF)/pDC-Irf8-ChIP-Seq(GSE66899)/Homer | 1e-185 | -4.280e+02 | 0.0000 | 1801.0 | 8.01% | 623.7 | 2.35% | motif file (matrix) | svg |
| 24 | G C T A C T G A T C G A A G T C A G T C C T G A A G T C G T C A C T G A T G C A | RUNX1(Runt)/Jurkat-RUNX1-ChIP-Seq(GSE29180)/Homer | 1e-114 | -2.629e+02 | 0.0000 | 3769.0 | 16.77% | 2601.7 | 9.82% | motif file (matrix) | svg |
| 25 | T A G C G C T A T C G A C T G A A G T C A G T C C T G A A G T C C G T A C T A G | RUNX(Runt)/HPC7-Runx1-ChIP-Seq(GSE22178)/Homer | 1e-96 | -2.219e+02 | 0.0000 | 2590.0 | 11.52% | 1642.7 | 6.20% | motif file (matrix) | svg |
| 26 | T G A C G C T A T C G A T G C A A G T C A G T C C G T A A G T C C G T A C T G A G C T A G T A C | RUNX2(Runt)/PCa-RUNX2-ChIP-Seq(GSE33889)/Homer | 1e-73 | -1.688e+02 | 0.0000 | 2895.0 | 12.88% | 2090.4 | 7.89% | motif file (matrix) | svg |
| 27 | A C T G G A T C G A C T A C T G A C G T C A T G A C T G A C G T A G C T C G A T | RUNX-AML(Runt)/CD4+-PolII-ChIP-Seq(Barski\_et\_al.)/Homer | 1e-67 | -1.550e+02 | 0.0000 | 2500.0 | 11.12% | 1766.3 | 6.67% | motif file (matrix) | svg |
| 28 | T G C A A G C T C T G A A T C G G A C T C T A G G T A C G A T C G T C A A G T C G T A C G A C T C T A G A T C G G C A T C A T G C A T G G A T C G T A C C T G A | CTCF(Zf)/CD4+-CTCF-ChIP-Seq(Barski\_et\_al.)/Homer | 1e-64 | -1.485e+02 | 0.0000 | 571.0 | 2.54% | 178.2 | 0.67% | motif file (matrix) | svg |
| 29 | T A G C A G T C T G A C A G T C C T A G A T C G A G T C C A T G T G A C A G T C G T A C A G T C A G T C G C A T C T A G A T C G G C A T A C T G A T C G G A T C | BORIS(Zf)/K562-CTCFL-ChIP-Seq(GSE32465)/Homer | 1e-49 | -1.144e+02 | 0.0000 | 650.0 | 2.89% | 281.0 | 1.06% | motif file (matrix) | svg |
| 30 | C A T G C T A G T C G A A C G T A C T G C G T A T A G C C G A T T G A C C G T A A G C T G A T C | Fra2(bZIP)/Striatum-Fra2-ChIP-Seq(GSE43429)/Homer | 1e-48 | -1.112e+02 | 0.0000 | 1480.0 | 6.58% | 974.8 | 3.68% | motif file (matrix) | svg |
| 31 | T C G A T C G A T A C G G A T C G T C A G T A C C G A T A G C T G T C A T G C A | Nkx3.1(Homeobox)/LNCaP-Nkx3.1-ChIP-Seq(GSE28264)/Homer | 1e-47 | -1.096e+02 | 0.0000 | 7464.0 | 33.21% | 7194.3 | 27.15% | motif file (matrix) | svg |
| 32 | C T A G T C G A A C G T A C T G C G T A A T G C A C G T G T A C C G T A A G C T G A T C G T A C | Atf3(bZIP)/GBM-ATF3-ChIP-Seq(GSE33912)/Homer | 1e-46 | -1.059e+02 | 0.0000 | 1993.0 | 8.87% | 1466.5 | 5.53% | motif file (matrix) | svg |
| 33 | A C T G C T A G T C G A C G A T C A T G G C T A A T C G C G A T G T A C G C T A A G C T G T A C | Fra1(bZIP)/BT549-Fra1-ChIP-Seq(GSE46166)/Homer | 1e-45 | -1.057e+02 | 0.0000 | 1679.0 | 7.47% | 1173.2 | 4.43% | motif file (matrix) | svg |
| 34 | C T G A T C A G G T A C G C T A A C T G T G A C G C A T C A T G | SCL(bHLH)/HPC7-Scl-ChIP-Seq(GSE13511)/Homer | 1e-45 | -1.049e+02 | 0.0000 | 11206.0 | 49.86% | 11503.4 | 43.41% | motif file (matrix) | svg |
| 35 | G A C T C T A G C T A G C T A G A C T G T C G A C T G A C T A G C T A G C T A G G T A C G T C A | ZNF467(Zf)/HEK293-ZNF467.GFP-ChIP-Seq(GSE58341)/Homer | 1e-43 | -9.981e+01 | 0.0000 | 2776.0 | 12.35% | 2257.9 | 8.52% | motif file (matrix) | svg |
| 36 | C T A G T C G A G C A T C A T G G C T A T A G C C G A T G T A C C T G A A G C T | JunB(bZIP)/DendriticCells-Junb-ChIP-Seq(GSE36099)/Homer | 1e-42 | -9.772e+01 | 0.0000 | 1666.0 | 7.41% | 1189.1 | 4.49% | motif file (matrix) | svg |
| 37 | C T A G T C G A C G A T A C T G C G T A T A C G A G C T T G A C G C T A A C G T G A T C T A G C | Fosl2(bZIP)/3T3L1-Fosl2-ChIP-Seq(GSE56872)/Homer | 1e-40 | -9.291e+01 | 0.0000 | 1070.0 | 4.76% | 666.7 | 2.52% | motif file (matrix) | svg |
| 38 | T C G A A C G T C A T G G C T A T A G C C G A T G T A C G C T A A C G T A T G C | AP-1(bZIP)/ThioMac-PU.1-ChIP-Seq(GSE21512)/Homer | 1e-39 | -9.140e+01 | 0.0000 | 2140.0 | 9.52% | 1667.5 | 6.29% | motif file (matrix) | svg |
| 39 | C A G T T G C A A C G T A C T G C G T A A T C G C G A T T G A C C G T A A C G T | BATF(bZIP)/Th17-BATF-ChIP-Seq(GSE39756)/Homer | 1e-39 | -9.100e+01 | 0.0000 | 1909.0 | 8.49% | 1445.3 | 5.45% | motif file (matrix) | svg |
| 40 | C T A G T C G A A C G T A C T G C G T A T A G C C G A T G T A C C G T A A G C T G A T C G T A C | Jun-AP1(bZIP)/K562-cJun-ChIP-Seq(GSE31477)/Homer | 1e-36 | -8.398e+01 | 0.0000 | 810.0 | 3.60% | 468.5 | 1.77% | motif file (matrix) | svg |
| 41 | T G A C C T G A C T A G T C G A C T G A A T G C C G T A A C T G G C A T G T A C G C A T A T C G G C A T A G C T G A T C | PR(NR)/T47D-PR-ChIP-Seq(GSE31130)/Homer | 1e-28 | -6.637e+01 | 0.0000 | 5898.0 | 26.24% | 5800.0 | 21.89% | motif file (matrix) | svg |
| 42 | C T G A T A C G G C A T A G C T A G C T A G T C T C G A A C T G C A G T A G C T A G C T G A T C | IRF3(IRF)/BMDM-Irf3-ChIP-Seq(GSE67343)/Homer | 1e-28 | -6.535e+01 | 0.0000 | 1263.0 | 5.62% | 933.4 | 3.52% | motif file (matrix) | svg |
| 43 | A G T C A C G T A C T G A G C T A C G T A C G T G T C A A G T C | Foxo1(Forkhead)/RAW-Foxo1-ChIP-Seq(Fan\_et\_al.)/Homer | 1e-26 | -6.135e+01 | 0.0000 | 5413.0 | 24.08% | 5308.2 | 20.03% | motif file (matrix) | svg |
| 44 | C T A G T C G A C T G A C G T A T A C G G A C T T C A G T C G A G T C A T G C A T A C G A G C T | IRF2(IRF)/Erythroblas-IRF2-ChIP-Seq(GSE36985)/Homer | 1e-21 | -4.957e+01 | 0.0000 | 451.0 | 2.01% | 255.7 | 0.97% | motif file (matrix) | svg |
| 45 | C G A T T A C G T G A C G A C T C A T G C G T A T A C G A C G T G T A C C T G A | Bach2(bZIP)/OCILy7-Bach2-ChIP-Seq(GSE44420)/Homer | 1e-20 | -4.821e+01 | 0.0000 | 598.0 | 2.66% | 384.8 | 1.45% | motif file (matrix) | svg |
| 46 | T G A C G C T A T G A C C G T A T C A G G A T C C G T A C A T G C A T G C T A G C T A G C T A G | Unknown-ESC-element(?)/mES-Nanog-ChIP-Seq(GSE11724)/Homer | 1e-20 | -4.806e+01 | 0.0000 | 1618.0 | 7.20% | 1361.2 | 5.14% | motif file (matrix) | svg |
| 47 | C T A G C T A G A G T C T C A G A C T G A C G T A C G T C T G A | MYB(HTH)/ERMYB-Myb-ChIPSeq(GSE22095)/Homer | 1e-19 | -4.592e+01 | 0.0000 | 4206.0 | 18.71% | 4121.5 | 15.55% | motif file (matrix) | svg |
| 48 | T G C A T A G C G A C T T G C A T G A C T G C A C G T A A G C T A G C T A G T C A G T C G T A C | GFY(?)/Promoter/Homer | 1e-17 | -4.117e+01 | 0.0000 | 280.0 | 1.25% | 137.4 | 0.52% | motif file (matrix) | svg |
| 49 | T A C G A T G C G C T A A C T G C G T A A C G T C G T A C T G A T A C G T C G A | Gata4(Zf)/Heart-Gata4-ChIP-Seq(GSE35151)/Homer | 1e-17 | -4.079e+01 | 0.0000 | 2804.0 | 12.47% | 2647.9 | 9.99% | motif file (matrix) | svg |
| 50 | C T A G A C T G C T A G T C A G T C A G T A C G C T A G A C T G | Maz(Zf)/HepG2-Maz-ChIP-Seq(GSE31477)/Homer | 1e-17 | -3.935e+01 | 0.0000 | 2901.0 | 12.91% | 2763.8 | 10.43% | motif file (matrix) | svg |
| 51 | C G T A C G T A C G T A G C A T G C A T T A C G G T A C G A C T A C T G C G T A A T C G A C G T G T A C C G T A A G C T | Bach1(bZIP)/K562-Bach1-ChIP-Seq(GSE31477)/Homer | 1e-15 | -3.652e+01 | 0.0000 | 218.0 | 0.97% | 99.9 | 0.38% | motif file (matrix) | svg |
| 52 | T C A G C T G A C G T A C G T A T A C G G C A T C T A G C T G A C G T A C G T A T A C G G A C T | IRF1(IRF)/PBMC-IRF1-ChIP-Seq(GSE43036)/Homer | 1e-15 | -3.530e+01 | 0.0000 | 640.0 | 2.85% | 467.0 | 1.76% | motif file (matrix) | svg |
| 53 | T C A G A G C T A T G C C G T A A G C T T C A G C A G T A C T G C T G A A G T C | MITF(bHLH)/MastCells-MITF-ChIP-Seq(GSE48085)/Homer | 1e-15 | -3.526e+01 | 0.0000 | 2213.0 | 9.85% | 2063.0 | 7.79% | motif file (matrix) | svg |
| 54 | G T C A G C A T A C T G G T A C G A C T A C T G G C T A A T C G C A G T G T A C C G T A A G C T | Nrf2(bZIP)/Lymphoblast-Nrf2-ChIP-Seq(GSE37589)/Homer | 1e-15 | -3.495e+01 | 0.0000 | 191.0 | 0.85% | 82.7 | 0.31% | motif file (matrix) | svg |
| 55 | A G C T A T G C G A C T G C A T C G T A A G C T G T A C C G A T A T C G A G T C | Gata6(Zf)/HUG1N-GATA6-ChIP-Seq(GSE51936)/Homer | 1e-14 | -3.374e+01 | 0.0000 | 2520.0 | 11.21% | 2402.6 | 9.07% | motif file (matrix) | svg |
| 56 | T A C G T C G A C A G T A C T G G C T A A T G C C G A T G T A C C G T A A C T G T A G C C G T A | NF-E2(bZIP)/K562-NFE2-ChIP-Seq(GSE31477)/Homer | 1e-14 | -3.253e+01 | 0.0000 | 216.0 | 0.96% | 105.8 | 0.40% | motif file (matrix) | svg |
| 57 | G A C T C T A G C T A G A G T C T G C A A C T G A C G T A C G T C T A G T C A G | AMYB(HTH)/Testes-AMYB-ChIP-Seq(GSE44588)/Homer | 1e-14 | -3.246e+01 | 0.0000 | 3643.0 | 16.21% | 3636.3 | 13.72% | motif file (matrix) | svg |
| 58 | C A G T T C A G G A T C A C T G A C G T C T A G A C T G A C T G G A C T C T A G | Egr1(Zf)/K562-Egr1-ChIP-Seq(GSE32465)/Homer | 1e-14 | -3.225e+01 | 0.0000 | 1377.0 | 6.13% | 1210.6 | 4.57% | motif file (matrix) | svg |
| 59 | T A C G T C A G A G C T A T G C C G T A A G T C T C A G A C G T A C T G T C G A | USF1(bHLH)/GM12878-Usf1-ChIP-Seq(GSE32465)/Homer | 1e-13 | -3.212e+01 | 0.0000 | 1044.0 | 4.64% | 873.9 | 3.30% | motif file (matrix) | svg |
| 60 | A G T C G C A T C G T A C G T A G T A C A C G T A C T G G A T C G A T C T C G A | BMYB(HTH)/Hela-BMYB-ChIP-Seq(GSE27030)/Homer | 1e-13 | -3.116e+01 | 0.0000 | 3565.0 | 15.86% | 3564.8 | 13.45% | motif file (matrix) | svg |
| 61 | T A G C G C T A A C T G C G T A A C G T C G T A C G T A T A C G T C A G T C G A | Gata1(Zf)/K562-GATA1-ChIP-Seq(GSE18829)/Homer | 1e-13 | -3.097e+01 | 0.0000 | 1657.0 | 7.37% | 1509.3 | 5.70% | motif file (matrix) | svg |
| 62 | A G C T A G T C A T G C A G C T A C G T C G T A A C G T A G T C C G A T A T G C | Gata2(Zf)/K562-GATA2-ChIP-Seq(GSE18829)/Homer | 1e-13 | -3.040e+01 | 0.0000 | 1865.0 | 8.30% | 1732.7 | 6.54% | motif file (matrix) | svg |
| 63 | G A C T C T A G G A T C C A G T A C T G C T G A A T G C G C A T A T G C C T G A | MafA(bZIP)/Islet-MafA-ChIP-Seq(GSE30298)/Homer | 1e-12 | -2.969e+01 | 0.0000 | 1819.0 | 8.09% | 1689.4 | 6.38% | motif file (matrix) | svg |
| 64 | C A T G T A C G T A G C G A T C G A T C A T G C G T A C G A C T T C A G A T G C C G A T A T C G C A G T A C T G G T A C | Zic3(Zf)/mES-Zic3-ChIP-Seq(GSE37889)/Homer | 1e-12 | -2.886e+01 | 0.0000 | 1361.0 | 6.06% | 1216.9 | 4.59% | motif file (matrix) | svg |
| 65 | A G C T C A T G G C A T G A T C T G C A C T A G G A T C A C G T | Tgif2(Homeobox)/mES-Tgif2-ChIP-Seq(GSE55404)/Homer | 1e-12 | -2.875e+01 | 0.0000 | 8168.0 | 36.34% | 8805.2 | 33.23% | motif file (matrix) | svg |
| 66 | A C G T T C G A T C G A A G T C G T C A T A C G A T G C A C G T A C T G A G C T | Myf5(bHLH)/GM-Myf5-ChIP-Seq(GSE24852)/Homer | 1e-12 | -2.869e+01 | 0.0000 | 1596.0 | 7.10% | 1462.0 | 5.52% | motif file (matrix) | svg |
| 67 | G C T A A T C G G C T A G A C T G C T A T C G A T A G C T C G A | GATA3(Zf)/iTreg-Gata3-ChIP-Seq(GSE20898)/Homer | 1e-12 | -2.829e+01 | 0.0000 | 4045.0 | 18.00% | 4128.2 | 15.58% | motif file (matrix) | svg |
| 68 | T C G A A C T G A C T G C G T A C G T A T C G A A G T C C T G A A T C G G T A C G C A T C A T G | ETS:E-box(ETS,bHLH)/HPC7-Scl-ChIP-Seq(GSE22178)/Homer | 1e-11 | -2.664e+01 | 0.0000 | 331.0 | 1.47% | 216.0 | 0.82% | motif file (matrix) | svg |
| 69 | G T C A T G C A G C T A A G T C C G T A A C T G T G A C G C A T T C A G C A G T | Ap4(bHLH)/AML-Tfap4-ChIP-Seq(GSE45738)/Homer | 1e-11 | -2.561e+01 | 0.0000 | 2794.0 | 12.43% | 2777.9 | 10.48% | motif file (matrix) | svg |
| 70 | C T G A A T G C G C T A G C A T A T G C C G T A T C G A C T G A C T A G T C A G T A C G G T C A | Tcf4(HMG)/Hct116-Tcf4-ChIP-Seq(SRA012054)/Homer | 1e-10 | -2.475e+01 | 0.0000 | 1372.0 | 6.10% | 1257.9 | 4.75% | motif file (matrix) | svg |
| 71 | A G C T A C G T A C T G A T G C A G T C C G T A C T G A T A C G | NF1-halfsite(CTF)/LNCaP-NF1-ChIP-Seq(Unpublished)/Homer | 1e-10 | -2.436e+01 | 0.0000 | 4261.0 | 18.96% | 4419.4 | 16.68% | motif file (matrix) | svg |
| 72 | C T G A T C A G A G T C C G T A A T C G A T G C C G A T A C T G A G T C G A C T A T C G A G T C | MyoD(bHLH)/Myotube-MyoD-ChIP-Seq(GSE21614)/Homer | 1e-10 | -2.366e+01 | 0.0000 | 1677.0 | 7.46% | 1588.4 | 5.99% | motif file (matrix) | svg |
| 73 | T C A G T G A C G T A C C G T A A C G T T G A C A C G T T C A G A G C T G A C T | NeuroD1(bHLH)/Islet-NeuroD1-ChIP-Seq(GSE30298)/Homer | 1e-10 | -2.337e+01 | 0.0000 | 1937.0 | 8.62% | 1869.3 | 7.05% | motif file (matrix) | svg |
| 74 | C T G A A T G C G C T A C G A T A T G C C G T A C G T A C G T A C T A G T A C G | Tcf3(HMG)/mES-Tcf3-ChIP-Seq(GSE11724)/Homer | 1e-10 | -2.331e+01 | 0.0000 | 744.0 | 3.31% | 622.9 | 2.35% | motif file (matrix) | svg |
| 75 | T A C G T A C G G T A C A T C G A C T G T A C G T C G A C T G A T C G A A T C G | E2F6(E2F)/Hela-E2F6-ChIP-Seq(GSE31477)/Homer | 1e-10 | -2.320e+01 | 0.0000 | 1034.0 | 4.60% | 917.9 | 3.46% | motif file (matrix) | svg |
| 76 | T C A G C G T A A G T C A G C T C G T A A G T C C T G A C G T A A G T C G C A T A G T C A G T C A G T C C T G A A C T G T G C A T C G A C A T G A T C G G A T C | Ronin(THAP)/ES-Thap11-ChIP-Seq(GSE51522)/Homer | 1e-9 | -2.301e+01 | 0.0000 | 106.0 | 0.47% | 41.6 | 0.16% | motif file (matrix) | svg |
| 77 | C T G A A T C G A G C T A G C T A C G T T A G C C T G A T A C G C G A T A C G T G A C T A G T C | ISRE(IRF)/ThioMac-LPS-Expression(GSE23622)/Homer | 1e-9 | -2.293e+01 | 0.0000 | 253.0 | 1.13% | 158.8 | 0.60% | motif file (matrix) | svg |
| 78 | T C G A A G T C C G T A A T C G A T G C C G A T A C T G A G T C A G C T A C T G | Tcf12(bHLH)/GM12878-Tcf12-ChIP-Seq(GSE32465)/Homer | 1e-9 | -2.282e+01 | 0.0000 | 2194.0 | 9.76% | 2152.6 | 8.12% | motif file (matrix) | svg |
| 79 | C A G T A C T G T C A G T G C A G C T A A T G C T C G A A T C G G T C A T G C A | ZNF189(Zf)/HEK293-ZNF189.GFP-ChIP-Seq(GSE58341)/Homer | 1e-9 | -2.165e+01 | 0.0000 | 2467.0 | 10.98% | 2462.8 | 9.29% | motif file (matrix) | svg |
| 80 | T C A G A C G T T C G A T A G C A G T C C G T A A C T G G T A C A C G T A C T G A T C G A G T C | Atoh1(bHLH)/Cerebellum-Atoh1-ChIP-Seq(GSE22111)/Homer | 1e-9 | -2.078e+01 | 0.0000 | 2521.0 | 11.22% | 2531.1 | 9.55% | motif file (matrix) | svg |
| 81 | C A T G A C T G C T A G T C G A T C G A T C G A T C G A T C A G T C A G T C A G T G A C T G A C C G T A A C T G T G C A C G A T A C T G | RBPJ:Ebox(?,bHLH)/Panc1-Rbpj1-ChIP-Seq(GSE47459)/Homer | 1e-8 | -1.946e+01 | 0.0000 | 918.0 | 4.08% | 823.1 | 3.11% | motif file (matrix) | svg |
| 82 | T C G A T C G A A G T C C G T A C T A G T A G C A C G T A C T G | MyoG(bHLH)/C2C12-MyoG-ChIP-Seq(GSE36024)/Homer | 1e-8 | -1.908e+01 | 0.0000 | 2341.0 | 10.42% | 2354.0 | 8.88% | motif file (matrix) | svg |
| 83 | C T G A T C A G C A G T C T A G A C T G C T A G G A T C A T C G A C T G C T G A T C A G G A T C | Sp5(Zf)/mES-Sp5.Flag-ChIP-Seq(GSE72989)/Homer | 1e-8 | -1.882e+01 | 0.0000 | 2175.0 | 9.68% | 2174.9 | 8.21% | motif file (matrix) | svg |
| 84 | G C T A G A C T G A C T T G C A C G T A A G T C C G T A T A G C G A T C G A C T | Eomes(T-box)/H9-Eomes-ChIP-Seq(GSE26097)/Homer | 1e-8 | -1.858e+01 | 0.0000 | 5863.0 | 26.08% | 6324.3 | 23.87% | motif file (matrix) | svg |
| 85 | C T A G C A G T T G A C C G T A G A T C T C A G G A C T C A T G | BMAL1(bHLH)/Liver-Bmal1-ChIP-Seq(GSE39860)/Homer | 1e-7 | -1.793e+01 | 0.0000 | 4113.0 | 18.30% | 4345.2 | 16.40% | motif file (matrix) | svg |
| 86 | C G A T T G C A T G C A G A T C C G T A A C T G T G A C G A C T C A T G A C T G | Tcf21(bHLH)/ArterySmoothMuscle-Tcf21-ChIP-Seq(GSE61369)/Homer | 1e-7 | -1.790e+01 | 0.0000 | 2159.0 | 9.61% | 2167.0 | 8.18% | motif file (matrix) | svg |
| 87 | T C G A C T A G A G T C A G T C C G T A C G T A A C G T T A G C T C A G T A C G | NFY(CCAAT)/Promoter/Homer | 1e-7 | -1.730e+01 | 0.0000 | 1617.0 | 7.19% | 1583.2 | 5.98% | motif file (matrix) | svg |
| 88 | A C G T T A C G G A T C A C T G A C G T C T A G A C T G A C T G G A T C C T A G C A T G C T A G | Egr2(Zf)/Thymocytes-Egr2-ChIP-Seq(GSE34254)/Homer | 1e-7 | -1.661e+01 | 0.0000 | 297.0 | 1.32% | 219.5 | 0.83% | motif file (matrix) | svg |
| 89 | T C A G A G C T A T G C C G T A A G T C T C A G A C G T A T C G T C G A A G T C G A T C T G A C | TFE3(bHLH)/MEF-TFE3-ChIP-Seq(GSE75757)/Homer | 1e-7 | -1.613e+01 | 0.0000 | 200.0 | 0.89% | 132.9 | 0.50% | motif file (matrix) | svg |
| 90 | A G T C A T C G C T A G A G C T G A C T C T A G A G T C A G T C G C T A C A G T T C A G T C A G G A T C C T G A T C G A G A T C | RFX(HTH)/K562-RFX3-ChIP-Seq(SRA012198)/Homer | 1e-6 | -1.552e+01 | 0.0000 | 182.0 | 0.81% | 118.3 | 0.45% | motif file (matrix) | svg |
| 91 | T C A G T A G C G A C T C A T G C T G A A T C G G C A T G T A C C G T A A C T G T A G C T G C A | MafK(bZIP)/C2C12-MafK-ChIP-Seq(GSE36030)/Homer | 1e-6 | -1.534e+01 | 0.0000 | 521.0 | 2.32% | 444.1 | 1.68% | motif file (matrix) | svg |
| 92 | C T A G A G C T G A C T C A T G A G T C A G T C G T C A C A G T C T A G T C A G G T A C C T G A T C G A G A T C T G A C | Rfx2(HTH)/LoVo-RFX2-ChIP-Seq(GSE49402)/Homer | 1e-6 | -1.511e+01 | 0.0000 | 203.0 | 0.90% | 138.7 | 0.52% | motif file (matrix) | svg |
| 93 | T C A G A C G T A G T C T C G A A G T C T C A G G C A T C T A G C T A G A G C T | Usf2(bHLH)/C2C12-Usf2-ChIP-Seq(GSE36030)/Homer | 1e-6 | -1.489e+01 | 0.0000 | 760.0 | 3.38% | 692.4 | 2.61% | motif file (matrix) | svg |
| 94 | A T G C G A T C G A C T A G C T C G A T C G A T G T C A C G A T T C G A A T C G T A G C T A G C | TATA-Box(TBP)/Promoter/Homer | 1e-6 | -1.473e+01 | 0.0000 | 3227.0 | 14.36% | 3397.5 | 12.82% | motif file (matrix) | svg |
| 95 | T C G A T G A C A G T C C G T A A G T C C T A G A C G T A C T G A C T G A G C T A G T C G C A T | Max(bHLH)/K562-Max-ChIP-Seq(GSE31477)/Homer | 1e-6 | -1.445e+01 | 0.0000 | 1381.0 | 6.14% | 1357.3 | 5.12% | motif file (matrix) | svg |
| 96 | C A T G C T A G A G C T G A C T C A T G A G T C G A T C G C T A C G A T C T A G T C A G G T A C C T G A T C G A | X-box(HTH)/NPC-H3K4me1-ChIP-Seq(GSE16256)/Homer | 1e-6 | -1.428e+01 | 0.0000 | 254.0 | 1.13% | 188.9 | 0.71% | motif file (matrix) | svg |
| 97 | C T A G T C G A C G A T C T A G G C A T C A G T C T A G G A T C C G T A G T C A | CEBP:AP1(bZIP)/ThioMac-CEBPb-ChIP-Seq(GSE21512)/Homer | 1e-6 | -1.422e+01 | 0.0000 | 1885.0 | 8.39% | 1910.7 | 7.21% | motif file (matrix) | svg |
| 98 | G A C T G C A T C T A G C G A T G A T C T C G A C A T G G A T C | Tgif1(Homeobox)/mES-Tgif1-ChIP-Seq(GSE55404)/Homer | 1e-6 | -1.419e+01 | 0.0000 | 7478.0 | 33.27% | 8272.8 | 31.22% | motif file (matrix) | svg |
| 99 | C G T A G A C T C G A T A T C G G T A C G C A T C A T G C G T A T A C G G C A T G T A C C G T A C A T G A T G C G C T A C T A G G C A T G C A T G C A T G A C T | MafB(bZIP)/BMM-Mafb-ChIP-Seq(GSE75722)/Homer | 1e-6 | -1.410e+01 | 0.0000 | 980.0 | 4.36% | 930.9 | 3.51% | motif file (matrix) | svg |
| 100 | T C G A T G A C G T A C C G T A C A G T T G A C A C G T A C T G A G C T A G C T | NeuroG2(bHLH)/Fibroblast-NeuroG2-ChIP-Seq(GSE75910)/Homer | 1e-6 | -1.398e+01 | 0.0000 | 3649.0 | 16.23% | 3885.2 | 14.66% | motif file (matrix) | svg |
| 101 | C G T A A T G C C G A T A C G T A G T C C G T A C G T A C G T A C T A G A T C G | TCFL2(HMG)/K562-TCF7L2-ChIP-Seq(GSE29196)/Homer | 1e-6 | -1.383e+01 | 0.0000 | 251.0 | 1.12% | 187.2 | 0.71% | motif file (matrix) | svg |
| 102 | A T G C A T C G T A C G A G C T A T C G C T G A A G T C C T A G A G C T A T G C C T G A A T G C | CRE(bZIP)/Promoter/Homer | 1e-5 | -1.379e+01 | 0.0000 | 376.0 | 1.67% | 308.3 | 1.16% | motif file (matrix) | svg |
| 103 | G A C T C G T A A C G T A C T G A G T C C G T A C T G A C G T A C A G T A C G T G T C A T C A G | Brn1(POU,Homeobox)/NPC-Brn1-ChIP-Seq(GSE35496)/Homer | 1e-5 | -1.366e+01 | 0.0000 | 656.0 | 2.92% | 593.0 | 2.24% | motif file (matrix) | svg |
| 104 | C G A T T G C A G T A C C G T A A G T C C T A G G A C T C A T G | NPAS(bHLH)/Liver-NPAS-ChIP-Seq(GSE39860)/Homer | 1e-5 | -1.342e+01 | 0.0000 | 3568.0 | 15.87% | 3803.9 | 14.36% | motif file (matrix) | svg |
| 105 | C T G A C T G A C T A G T C G A C G T A A T G C C G T A A C T G C G T A A C G T C T G A C G A T A G C T C G T A A C G T A G T C C G A T T A C G G T C A G C A T | GATA(Zf),IR3/iTreg-Gata3-ChIP-Seq(GSE20898)/Homer | 1e-5 | -1.338e+01 | 0.0000 | 438.0 | 1.95% | 373.0 | 1.41% | motif file (matrix) | svg |
| 106 | T G A C C T A G A C T G T A G C C G A T A C T G A T G C C A T G A T C G A T C G A T C G T A G C C T G A T A G C G C T A A C T G C G T A A G C T C G T A C T G A | GATA:SCL(Zf,bHLH)/Ter119-SCL-ChIP-Seq(GSE18720)/Homer | 1e-5 | -1.302e+01 | 0.0000 | 315.0 | 1.40% | 252.9 | 0.95% | motif file (matrix) | svg |
| 107 | A G T C G T A C A G C T C T A G A G T C C G A T A C T G C G T A A C T G G T C A | Zic(Zf)/Cerebellum-ZIC1.2-ChIP-Seq(GSE60731)/Homer | 1e-5 | -1.288e+01 | 0.0000 | 1789.0 | 7.96% | 1821.8 | 6.88% | motif file (matrix) | svg |
| 108 | C T G A T G A C T G A C C G T A A C G T T G A C A G C T C T A G A C G T G A C T | Olig2(bHLH)/Neuron-Olig2-ChIP-Seq(GSE30882)/Homer | 1e-5 | -1.239e+01 | 0.0000 | 4614.0 | 20.53% | 5012.2 | 18.92% | motif file (matrix) | svg |
| 109 | T C G A C A T G C A T G T A C G G T A C T C G A A G T C C A G T C T G A C G T A A G T C G A C T | ZNF264(Zf)/HEK293-ZNF264.GFP-ChIP-Seq(GSE58341)/Homer | 1e-5 | -1.205e+01 | 0.0000 | 1288.0 | 5.73% | 1282.8 | 4.84% | motif file (matrix) | svg |
| 110 | A T G C T C G A A G T C A G C T A C G T G T A C A G T C G C T A C T A G C A T G G T C A C T G A T C A G A G T C | Stat3+il21(Stat)/CD4-Stat3-ChIP-Seq(GSE19198)/Homer | 1e-5 | -1.198e+01 | 0.0000 | 1762.0 | 7.84% | 1803.2 | 6.81% | motif file (matrix) | svg |
| 111 | T C G A A C G T A C T G C T G A A G T C T C A G A G C T G T A C C G T A A G C T G A T C T C G A | JunD(bZIP)/K562-JunD-ChIP-Seq/Homer | 1e-5 | -1.184e+01 | 0.0000 | 187.0 | 0.83% | 135.4 | 0.51% | motif file (matrix) | svg |
| 112 | C T G A C T A G T G C A C T G A T C G A A G C T C A T G T C G A A G T C G A C T A C G T A G T C G A T C G A T C G A C T | ZNF528(Zf)/HEK293-ZNF528.GFP-ChIP-Seq(GSE58341)/Homer | 1e-5 | -1.181e+01 | 0.0000 | 36.0 | 0.16% | 10.2 | 0.04% | motif file (matrix) | svg |
| 113 | A C T G T C A G A G C T G A C T C A T G A G T C A G T C G C T A C G A T C T A G T C A G G T A C C T G A T C G A | Rfx1(HTH)/NPC-H3K4me1-ChIP-Seq(GSE16256)/Homer | 1e-5 | -1.179e+01 | 0.0000 | 460.0 | 2.05% | 404.1 | 1.53% | motif file (matrix) | svg |
| 114 | C G T A C G T A A G C T G A C T T G C A G T C A A C G T A G C T C T G A T C A G | Lhx3(Homeobox)/Neuron-Lhx3-ChIP-Seq(GSE31456)/Homer | 1e-5 | -1.156e+01 | 0.0000 | 3798.0 | 16.90% | 4098.2 | 15.47% | motif file (matrix) | svg |
| 115 | T C G A C G T A A G T C A G C T C G T A A G T C T C G A G C T A G A C T C G A T A G T C A G T C A G T C C T G A T C A G T G C A T C G A C A G T A T C G A G T C | GFY-Staf(?,Zf)/Promoter/Homer | 1e-5 | -1.154e+01 | 0.0000 | 142.0 | 0.63% | 95.7 | 0.36% | motif file (matrix) | svg |
| 116 | A C T G A C G T C A T G A T C G A T C G T G A C A C T G A T C G A T C G T G C A C T G A C G T A | E2F3(E2F)/MEF-E2F3-ChIP-Seq(GSE71376)/Homer | 1e-4 | -1.084e+01 | 0.0001 | 1017.0 | 4.52% | 1001.3 | 3.78% | motif file (matrix) | svg |
| 117 | T A C G T C G A T A G C A G T C C G T A A G T C C T A G G C A T A C T G A T C G | n-Myc(bHLH)/mES-nMyc-ChIP-Seq(GSE11431)/Homer | 1e-4 | -1.067e+01 | 0.0001 | 1319.0 | 5.87% | 1332.4 | 5.03% | motif file (matrix) | svg |
| 118 | T C G A T G A C G C A T A G C T C A G T G A T C G C T A G A T C G A C T A C G T G C A T A G T C | PRDM1(Zf)/Hela-PRDM1-ChIP-Seq(GSE31477)/Homer | 1e-4 | -1.064e+01 | 0.0001 | 1841.0 | 8.19% | 1909.1 | 7.20% | motif file (matrix) | svg |
| 119 | A T G C A T G C A T C G T A C G A G C T A G T C G C T A A G T C T C A G G A C T A C T G T C G A | E-box(bHLH)/Promoter/Homer | 1e-4 | -1.044e+01 | 0.0001 | 150.0 | 0.67% | 106.2 | 0.40% | motif file (matrix) | svg |
| 120 | C G A T C T A G C T G A A T G C C T G A T C G A C G T A C T G A T C G A T A G C A G T C C G T A A C T G T C G A A T G C | Hand2(bHLH)/Mesoderm-Hand2-ChIP-Seq(GSE61475)/Homer | 1e-4 | -9.897e+00 | 0.0002 | 1231.0 | 5.48% | 1245.1 | 4.70% | motif file (matrix) | svg |
| 121 | C T A G T A C G G A T C G T A C G C T A A G C T A G C T G T C A T C G A T A G C | Nanog(Homeobox)/mES-Nanog-ChIP-Seq(GSE11724)/Homer | 1e-4 | -9.601e+00 | 0.0002 | 9727.0 | 43.28% | 11012.2 | 41.56% | motif file (matrix) | svg |
| 122 | C A G T T C A G A G C T G A C T A C G T A G T C G A T C G A C T C T G A A C T G G A T C C G T A C T G A A G T C G T A C | Rfx6(HTH)/Min6b1-Rfx6.HA-ChIP-Seq(GSE62844)/Homer | 1e-4 | -9.451e+00 | 0.0002 | 2289.0 | 10.18% | 2430.0 | 9.17% | motif file (matrix) | svg |
| 123 | C T A G T C A G C A G T T C A G A C T G A C T G G A T C C T A G A C T G C T A G T C A G A T G C | KLF14(Zf)/HEK293-KLF14.GFP-ChIP-Seq(GSE58341)/Homer | 1e-4 | -9.447e+00 | 0.0002 | 4006.0 | 17.82% | 4379.5 | 16.53% | motif file (matrix) | svg |
| 124 | A T G C A G T C G C T A C G A T C G T A G C A T G C T A C G A T C T A G C A T G T G A C G T C A | CArG(MADS)/PUER-Srf-ChIP-Seq(Sullivan\_et\_al.)/Homer | 1e-4 | -9.381e+00 | 0.0002 | 762.0 | 3.39% | 741.5 | 2.80% | motif file (matrix) | svg |
| 125 | G C A T C G T A C G A T A C T G A G T C G C T A C T G A C G T A C A G T A C T G C G T A T C A G | Oct6(POU,Homeobox)/NPC-Pou3f1-ChIP-Seq(GSE35496)/Homer | 1e-3 | -9.133e+00 | 0.0003 | 904.0 | 4.02% | 897.3 | 3.39% | motif file (matrix) | svg |
| 126 | T C A G G A C T C A G T C T G A A G C T C T A G G A C T T G C A C T G A A G T C | HLF(bZIP)/HSC-HLF.Flag-ChIP-Seq(GSE69817)/Homer | 1e-3 | -8.977e+00 | 0.0004 | 1939.0 | 8.63% | 2044.9 | 7.72% | motif file (matrix) | svg |
| 127 | C T G A C T A G T C G A C G T A A T G C C G T A A T C G C G A T T A G C G C A T A T C G G C A T A G C T G A T C G A C T A G C T | ARE(NR)/LNCAP-AR-ChIP-Seq(GSE27824)/Homer | 1e-3 | -8.798e+00 | 0.0004 | 565.0 | 2.51% | 536.6 | 2.03% | motif file (matrix) | svg |
| 128 | T A C G T C A G T G C A A G C T T G A C A G C T A G T C A C T G G A T C A C T G T C G A A C T G C T G A C T G A A T G C | ZBTB33(Zf)/GM12878-ZBTB33-ChIP-Seq(GSE32465)/Homer | 1e-3 | -8.741e+00 | 0.0005 | 56.0 | 0.25% | 29.3 | 0.11% | motif file (matrix) | svg |
| 129 | C G T A A C G T A G C T C G A T C T A G G T A C C G T A A G C T C G T A G C T A | Oct4(POU,Homeobox)/mES-Oct4-ChIP-Seq(GSE11431)/Homer | 1e-3 | -8.612e+00 | 0.0005 | 1025.0 | 4.56% | 1035.8 | 3.91% | motif file (matrix) | svg |
| 130 | G T C A G C A T G C T A C A G T C T A G G A T C C G T A C T G A C G T A C G A T | Oct2(POU,Homeobox)/Bcell-Oct2-ChIP-Seq(GSE21512)/Homer | 1e-3 | -8.479e+00 | 0.0006 | 613.0 | 2.73% | 590.6 | 2.23% | motif file (matrix) | svg |
| 131 | A T G C G A C T A C T G C A G T G A T C A C G T T A C G T A C G | Smad2(MAD)/ES-SMAD2-ChIP-Seq(GSE29422)/Homer | 1e-3 | -8.415e+00 | 0.0006 | 3256.0 | 14.49% | 3545.8 | 13.38% | motif file (matrix) | svg |
| 132 | C T A G G C A T G A T C C G T A A G T C T C A G G A C T C T A G | CLOCK(bHLH)/Liver-Clock-ChIP-Seq(GSE39860)/Homer | 1e-3 | -8.332e+00 | 0.0007 | 1010.0 | 4.49% | 1022.9 | 3.86% | motif file (matrix) | svg |
| 133 | C T A G A G T C T A C G T A C G T G A C C G T A A C T G T A G C G C A T C A T G A T G C A G C T | Ascl1(bHLH)/NeuralTubes-Ascl1-ChIP-Seq(GSE55840)/Homer | 1e-3 | -8.317e+00 | 0.0007 | 3516.0 | 15.64% | 3844.8 | 14.51% | motif file (matrix) | svg |
| 134 | T G C A A T G C A C G T A C G T A C G T A T G C C T A G A C G T A C G T A G C T G A T C A G C T | T1ISRE(IRF)/ThioMac-Ifnb-Expression/Homer | 1e-3 | -8.033e+00 | 0.0009 | 40.0 | 0.18% | 18.6 | 0.07% | motif file (matrix) | svg |
| 135 | C G A T C T A G T C G A A G C T C G A T C T G A C G T A A G C T A C T G C T A G A T G C G A T C | Hoxb4(Homeobox)/ES-Hoxb4-ChIP-Seq(GSE34014)/Homer | 1e-3 | -7.920e+00 | 0.0010 | 415.0 | 1.85% | 385.4 | 1.45% | motif file (matrix) | svg |
| 136 | T A C G A T C G T A G C G A T C A C T G A C G T A G T C A C G T C T A G A T C G | Smad4(MAD)/ESC-SMAD4-ChIP-Seq(GSE29422)/Homer | 1e-3 | -7.828e+00 | 0.0011 | 3270.0 | 14.55% | 3574.8 | 13.49% | motif file (matrix) | svg |
| 137 | A T G C C T G A A T C G T A C G A G T C C G A T T C A G C G A T C T A G A G C T G T C A G T C A C G T A A G T C C G T A T A C G C T G A | Fox:Ebox(Forkhead,bHLH)/Panc1-Foxa2-ChIP-Seq(GSE47459)/Homer | 1e-3 | -7.730e+00 | 0.0012 | 2132.0 | 9.49% | 2283.9 | 8.62% | motif file (matrix) | svg |
| 138 | T A C G T A C G G T A C A T C G T A C G T A C G G T C A C T G A C G T A G A C T | E2F4(E2F)/K562-E2F4-ChIP-Seq(GSE31477)/Homer | 1e-3 | -7.643e+00 | 0.0013 | 557.0 | 2.48% | 539.0 | 2.03% | motif file (matrix) | svg |
| 139 | A T G C A G T C G T A C A G C T T C G A C T A G G A T C C T G A G T C A A G T C G C T A T C A G | Rfx5(HTH)/GM12878-Rfx5-ChIP-Seq(GSE31477)/Homer | 1e-3 | -7.640e+00 | 0.0013 | 735.0 | 3.27% | 730.4 | 2.76% | motif file (matrix) | svg |
| 140 | G A C T G T A C T G C A A C G T G A T C G C T A T C G A A C G T A G T C C G T A | Pdx1(Homeobox)/Islet-Pdx1-ChIP-Seq(SRA008281)/Homer | 1e-3 | -7.403e+00 | 0.0016 | 2115.0 | 9.41% | 2270.1 | 8.57% | motif file (matrix) | svg |
| 141 | A T G C G A T C C G A T A C G T A C G T A C T G C A G T A G C T | Sox3(HMG)/NPC-Sox3-ChIP-Seq(GSE33059)/Homer | 1e-3 | -7.322e+00 | 0.0017 | 4188.0 | 18.63% | 4639.2 | 17.51% | motif file (matrix) | svg |
| 142 | A T G C A G T C G A T C C G T A A C G T A C G T A C T G A C G T A G C T G A T C | Sox2(HMG)/mES-Sox2-ChIP-Seq(GSE11431)/Homer | 1e-3 | -7.242e+00 | 0.0018 | 2022.0 | 9.00% | 2168.2 | 8.18% | motif file (matrix) | svg |
| 143 | C G T A C T A G C A T G A G C T A C T G C G A T A T C G C G T A G T C A G T C A | Tbet(T-box)/CD8-Tbet-ChIP-Seq(GSE33802)/Homer | 1e-3 | -7.174e+00 | 0.0020 | 2925.0 | 13.01% | 3195.7 | 12.06% | motif file (matrix) | svg |
| 144 | C G A T T A C G G A T C G C T A T C A G G A C T C G A T G T A C G A T C G T C A T C G A T G A C C G T A C T A G G A C T C T A G C T A G G T A C A G T C C G T A | CTCF-SatelliteElement(Zf?)/CD4+-CTCF-ChIP-Seq(Barski\_et\_al.)/Homer | 1e-3 | -7.158e+00 | 0.0020 | 48.0 | 0.21% | 26.1 | 0.10% | motif file (matrix) | svg |
| 145 | T C A G T C A G T A G C A G T C C T G A A G T C C T A G A C G T A C T G A T C G | c-Myc(bHLH)/mES-cMyc-ChIP-Seq(GSE11431)/Homer | 1e-3 | -7.043e+00 | 0.0022 | 935.0 | 4.16% | 956.8 | 3.61% | motif file (matrix) | svg |
| 146 | T G A C C T G A C T A G C T G A C G T A A G T C C T G A A C G T G C A T T A G C G C A T A T C G G A C T G A C T G A T C | GRE(NR),IR3/RAW264.7-GRE-ChIP-Seq(Unpublished)/Homer | 1e-2 | -6.905e+00 | 0.0025 | 514.0 | 2.29% | 499.7 | 1.89% | motif file (matrix) | svg |
| 147 | A G C T A G C T T A G C A T C G A G T C A C T G A T G C A T C G T C G A C T G A T C G A C T G A | E2F(E2F)/Hela-CellCycle-Expression/Homer | 1e-2 | -6.838e+00 | 0.0027 | 59.0 | 0.26% | 36.7 | 0.14% | motif file (matrix) | svg |
| 148 | A G C T G C A T A C T G A C G T A G T C A C G T C T A G T A C G | Smad3(MAD)/NPC-Smad3-ChIP-Seq(GSE36673)/Homer | 1e-2 | -6.789e+00 | 0.0028 | 6490.0 | 28.87% | 7319.3 | 27.62% | motif file (matrix) | svg |
| 149 | T G C A A G C T C A T G C G T A A G C T A C T G G A T C G T C A C G T A A G C T | Atf4(bZIP)/MEF-Atf4-ChIP-Seq(GSE35681)/Homer | 1e-2 | -6.719e+00 | 0.0030 | 674.0 | 3.00% | 674.6 | 2.55% | motif file (matrix) | svg |
| 150 | A G T C C G A T C T G A C G T A A C G T C A G T T C A G T G A C | Isl1(Homeobox)/Neuron-Isl1-ChIP-Seq(GSE31456)/Homer | 1e-2 | -5.985e+00 | 0.0061 | 4567.0 | 20.32% | 5114.8 | 19.30% | motif file (matrix) | svg |
| 151 | A G C T G T C A C G T A A C G T A C G T C T G A T C A G A T G C | Lhx2(Homeobox)/HFSC-Lhx2-ChIP-Seq(GSE48068)/Homer | 1e-2 | -5.958e+00 | 0.0062 | 2321.0 | 10.33% | 2534.5 | 9.57% | motif file (matrix) | svg |
| 152 | T C G A G A C T A G C T T G A C A G C T G T A C T C A G G A T C A T C G T G C A A C T G C T G A | GFX(?)/Promoter/Homer | 1e-2 | -5.926e+00 | 0.0064 | 31.0 | 0.14% | 15.8 | 0.06% | motif file (matrix) | svg |
| 153 | A C T G C A G T A C G T C G T A C G T A A C G T A C T G C T G A | Nkx6.1(Homeobox)/Islet-Nkx6.1-ChIP-Seq(GSE40975)/Homer | 1e-2 | -5.724e+00 | 0.0078 | 5947.0 | 26.46% | 6723.4 | 25.37% | motif file (matrix) | svg |
| 154 | T A C G A T G C G A C T A C T G A G C T A G T C G T C A T G C A A C G T A G T C G C T A T G C A | Pknox1(Homeobox)/ES-Prep1-ChIP-Seq(GSE63282)/Homer | 1e-2 | -5.546e+00 | 0.0092 | 478.0 | 2.13% | 474.0 | 1.79% | motif file (matrix) | svg |
| 155 | C A T G G C A T C G A T C G T A C G T A G A T C A G T C A G C T C G T A C G T A A C G T A G T C C G T A C G T A G C A T | DUX4(Homeobox)/Myoblasts-DUX4.V5-ChIP-Seq(GSE75791)/Homer | 1e-2 | -5.538e+00 | 0.0092 | 83.0 | 0.37% | 62.1 | 0.23% | motif file (matrix) | svg |
| 156 | C T A G C G T A G T C A C G T A A G T C G A T C A G C T C T A G C G T A A C G T G T C A G A T C | Six2(Homeobox)/NephronProgenitor-Six2-ChIP-Seq(GSE39837)/Homer | 1e-2 | -5.481e+00 | 0.0097 | 2320.0 | 10.32% | 2544.6 | 9.60% | motif file (matrix) | svg |
| 157 | C G T A C T G A C T A G C G T A C G T A A G T C C G T A C A G T G C A T G T C A C G A T A C T G A C G T G C A T G A T C | PGR(NR)/EndoStromal-PGR-ChIP-Seq(GSE69539)/Homer | 1e-2 | -5.470e+00 | 0.0098 | 537.0 | 2.39% | 539.1 | 2.03% | motif file (matrix) | svg |
| 158 | T G C A G C A T C G A T C G T A C A G T A C T G G T A C C G T A C T G A A G C T G T C A A C T G C T A G G T C A C G A T A C T G G T A C T G C A C G T A A G C T | CEBP:CEBP(bZIP)/MEF-Chop-ChIP-Seq(GSE35681)/Homer | 1e-2 | -5.460e+00 | 0.0098 | 378.0 | 1.68% | 367.5 | 1.39% | motif file (matrix) | svg |
| 159 | A T G C C G T A A C T G C G T A A C G T G C T A T C G A A G C T C G A T C G T A A C G T A G T C C G A T A C T G G A T C | GATA(Zf),IR4/iTreg-Gata3-ChIP-Seq(GSE20898)/Homer | 1e-2 | -5.443e+00 | 0.0099 | 191.0 | 0.85% | 170.0 | 0.64% | motif file (matrix) | svg |
| 160 | C T G A C T G A C A T G A T C G A G C T A T C G G A C T C A T G C T G A G T C A | Tbr1(T-box)/Cortex-Tbr1-ChIP-Seq(GSE71384)/Homer | 1e-2 | -5.379e+00 | 0.0105 | 3437.0 | 15.29% | 3828.4 | 14.45% | motif file (matrix) | svg |
| 161 | A T G C A G C T T C A G T G A C T C A G A T G C T G C A A C G T A T C G G A T C A C T G A G T C | NRF1(NRF)/MCF7-NRF1-ChIP-Seq(Unpublished)/Homer | 1e-2 | -5.233e+00 | 0.0121 | 69.0 | 0.31% | 50.4 | 0.19% | motif file (matrix) | svg |
| 162 | G C T A T A G C A G C T A T C G G T C A C G T A G C T A A T G C G A T C C T G A | IRF4(IRF)/GM12878-IRF4-ChIP-Seq(GSE32465)/Homer | 1e-2 | -5.231e+00 | 0.0121 | 1144.0 | 5.09% | 1216.2 | 4.59% | motif file (matrix) | svg |
| 163 | C T G A C G A T C T A G T C A G G A T C C T G A T C A G G A T C C T G A A C T G A G T C G C T A A C G T A G T C G C A T | PRDM9(Zf)/Testis-DMC1-ChIP-Seq(GSE35498)/Homer | 1e-2 | -5.167e+00 | 0.0127 | 1011.0 | 4.50% | 1068.8 | 4.03% | motif file (matrix) | svg |
| 164 | T A G C G T A C A G T C G T A C C G A T A G T C A G T C A G T C A G T C A G T C C G T A G A T C | Zfp281(Zf)/ES-Zfp281-ChIP-Seq(GSE81042)/Homer | 1e-2 | -5.148e+00 | 0.0129 | 403.0 | 1.79% | 397.4 | 1.50% | motif file (matrix) | svg |
| 165 | T A C G C T G A C T A G T C G A C G T A A G T C C T G A A T C G G C A T T A G C G A C T A C T G A C G T A G C T A G T C G A C T | GRE(NR),IR3/A549-GR-ChIP-Seq(GSE32465)/Homer | 1e-2 | -5.112e+00 | 0.0133 | 303.0 | 1.35% | 290.8 | 1.10% | motif file (matrix) | svg |
| 166 | C T A G A T G C A T G C C G A T A C T G G A C T A T G C G C T A T G A C A G C T T A G C G C T A | PBX1(Homeobox)/MCF7-PBX1-ChIP-Seq(GSE28007)/Homer | 1e-2 | -5.076e+00 | 0.0137 | 177.0 | 0.79% | 159.0 | 0.60% | motif file (matrix) | svg |
| 167 | T C A G C A T G C A T G A C T G A C T G A G C T A C T G A C G T A C T G C A G T A T G C A G T C | KLF10(Zf)/HEK293-KLF10.GFP-ChIP-Seq(GSE58341)/Homer | 1e-2 | -5.033e+00 | 0.0142 | 1156.0 | 5.14% | 1233.0 | 4.65% | motif file (matrix) | svg |
| 168 | A C T G C T A G G A T C C G T A C T A G A G T C T G C A G A C T C G T A A C G T T C A G A G T C A C G T C T G A A G T C A G T C G A T C C G T A T C A G T A C G | EBNA1(EBV-virus)/Raji-EBNA1-ChIP-Seq(GSE30709)/Homer | 1e-2 | -4.963e+00 | 0.0151 | 45.0 | 0.20% | 29.2 | 0.11% | motif file (matrix) | svg |
| 169 | A G T C C G A T A C T G A T C G T G A C G C T A C A T G A T C G T G A C C G A T A C T G T A G C G T A C G T C A | Tlx?(NR)/NPC-H3K4me1-ChIP-Seq(GSE16256)/Homer | 1e-2 | -4.769e+00 | 0.0183 | 897.0 | 3.99% | 947.1 | 3.57% | motif file (matrix) | svg |
| 170 | G T A C C A G T A C T G A C T G A C T G G A T C A C T G A C G T A C T G A C T G A G T C G A T C | KLF6(Zf)/PDAC-KLF6-ChIP-Seq(GSE64557)/Homer | 1e-2 | -4.723e+00 | 0.0190 | 1993.0 | 8.87% | 2189.3 | 8.26% | motif file (matrix) | svg |
| 171 | T C G A G C A T A C G T C T A G G T A C T C G A G C A T T G A C T C G A A C G T | Chop(bZIP)/MEF-Chop-ChIP-Seq(GSE35681)/Homer | 1e-2 | -4.716e+00 | 0.0191 | 556.0 | 2.47% | 569.4 | 2.15% | motif file (matrix) | svg |
| 172 | T G A C T C G A C T G A C T G A A T G C G A T C C T A G T A C G G A C T G A C T G A T C T C G A C T G A C T G A A T G C G A T C C T A G A T C G G A C T G A C T | Tcfcp2l1(CP2)/mES-Tcfcp2l1-ChIP-Seq(GSE11431)/Homer | 1e-2 | -4.631e+00 | 0.0206 | 237.0 | 1.05% | 224.1 | 0.85% | motif file (matrix) | svg |
| 173 | C T A G T A C G A G T C G T A C T C G A A C G T G T C A G C T A G C T A C G A T A G T C G C T A | HOXA9(Homeobox)/HSC-Hoxa9-ChIP-Seq(GSE33509)/Homer | 1e-2 | -4.621e+00 | 0.0207 | 1426.0 | 6.34% | 1546.5 | 5.84% | motif file (matrix) | svg |
